# Apple Pomace as a Sustainable Substrate in Sourdough Fermentation

**DOI:** 10.3389/fmicb.2021.742020

**Published:** 2021-12-15

**Authors:** Gheorghe Adrian Martău, Bernadette-Emőke Teleky, Floricuţa Ranga, Ioana Delia Pop, Dan Cristian Vodnar

**Affiliations:** ^1^Institute of Life Sciences, University of Agricultural Sciences and Veterinary Medicine, Cluj-Napoca, Romania; ^2^Faculty of Food Science and Technology, University of Agricultural Sciences and Veterinary Medicine, Cluj-Napoca, Romania; ^3^Department of Land Measurements and Exact Sciences, Horticulture Faculty, University of Agricultural Sciences and Veterinary Medicine, Cluj-Napoca, Romania

**Keywords:** waste management, apple by-product, wheat flour, *Fructilactobacillus florum*, *Saccharomyces cerevisiae*, circular economy

## Abstract

Innovations range from food production, land use, and emissions all the way to improved diets and waste management. Global apple production has amounted to over 87 million tons/year, while 18% are processed, resulting in 20–35% (apple fruit fresh weight) apple pomace (AP). Using modern AP management, integrated knowledge in innovative fermentation demonstrates opportunities for reducing environmental pollution and integration into a circular economy. With this association in view, integrating AP flour during sourdough fermentation increases the nutritional value, highlighting a new approach that could guide innovative fermented foods. In this study, the wheat flour (WF) and AP flour were mixed at different ratios, hydrated with water (1:1 *w*/*v*), and fermented using a selective culture of *Fructilactobacillus florum* DSM 22689 and baker’s yeast (single and co-culture). Sourdough fermentation was monitored and analyzed for 72 h. Results suggested that AP may be an important source of organic acids and fermentable sugars that increase nutritional sourdough value. AP flour addition in WF had a positive effect, especially in fermentations with 95% WF and 5% AP, mainly in co-culture fermentation.

## Introduction

Food waste and by-products are a severe global problem, especially in many developed countries (Gu et al., 2019). Additionally, food demand has increased due to urbanization, population growth, and income growth, and meeting its sustainability remains a major global challenge in the long run (Hu et al., 2020; Szabo et al., 2021). One of the most concerning industries is apple juice, of which production generates a massive volume of waste, considering the annual processed tonnage of up to 12 million tons (Mt) (Rabetafika et al., 2014). By contrast, the low cost and high abundance of this waste highlight the economical perspectives of its potentially valuable components (Iriondo-DeHond et al., 2018).

**FIGURE 1:**
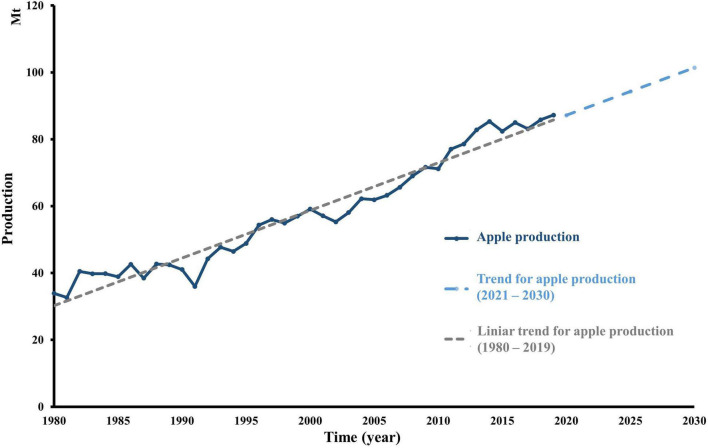
Evolution of world apple production over time. Apple global production (tons) from 1980 until 2019, according to FAO, and TREND production until 2030. The trendline was calculated considering apple production every year from 1980 to 2019. The TREND function returns values along with a linear trend. It fits a straight line (using the method of least squares) to the array’s known_y’s and known_x’s. TREND returns the *y*-values along that line for the array of new_x’s that you specify.

Apple (*Malus* sp.) is among the most popular fruits in the world. Global production has over 87 Mt in 2019 compared to 1990, where just over 47 Mt were produced (FAO, 2021). In addition, global production is expected to undergo a constant increase in the following years (Spengler, 2019; Puric et al., 2020). In case apple production and consumption will exhibit the same trend and constant growth, it will increase by 16%, more precisely over 14.17 Mt until 2030 (Figure 1). Additionally, 18% of global production is processed, which generates a significant volume of by-products (Rabetafika et al., 2014). Apple pomace (AP) is the pressed by-product obtained after apple processing, including juice, cider, wine, distilled spirit, vinegar manufacturing, and jelly industrial processes. The solid pomace represents 20–35% of the fresh weight of the apple fruit. As such, the residue is composed of a mixture of peel, core, seed, calyx, stem, and pulp. A significant fraction comes from the epi-mesocarp, accounting for 95.5% of the solid waste (Giovanetti Canteri et al., 2012). The AP composition consists of 94.5% flesh and skin, 4.1% seeds, and 1.1% fruit stems (Candrawinata et al., 2013; Gunes et al., 2019). By contrast, apple seeds contain proteins and oils in huge quantities, respectively, up to 49.5 and 24%. In addition, apple seeds contain a cyanogenic glycoside, amygdalin, and the degradation of which by β-glucosidase naturally present in the human intestine can lead to cyanide formation, causing severe human toxicity. Bolarinwa et al. (2015) have quantified amygdalin contents of seeds from 15 varieties of apples and revealed that it ranged from 1 to 4 mg/g. Alike, because the boiling point of hydrogen cyanide is 26°C, it easily volatilizes during food processing. However, the significant AP component is constituted by the dietary fiber of around 65% of dry weight. The insoluble fiber represents the major dietary fiber in all pomace. Cellulose is an essential fraction attaining 43% of the pomace, while hemicellulose is the second most important fiber in AP (19.9–32.2%) (Rupasinghe et al., 2007). The phenolic compounds are concentrated in the seed and peel by-products, principally as chlorogenic acid and phloridzin (Rabetafika et al., 2014).

**FIGURE 2:**
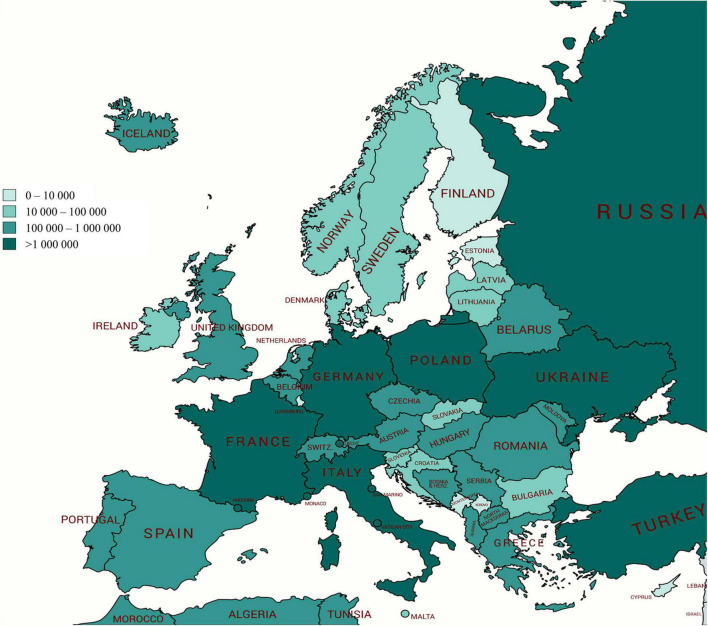
Production of apples (tons) in Europe for every country in 2018; Poland, Italy, and France are the most important producers (http://www.fao.org/faostat).

Various technologies are applied to apple fruits that influence by-product composition and ultimately product composition. During juice and cider manufacturing, processes may vary from one company to another. The continuous press system is the most applied in industrial fruit juice, but in small companies, a discontinuous vertical hydraulic press can be used (Heloísa et al., 2007). In Europe, over 17 Mt of apples are produced every year, and Poland, Italy, and France are the most important producers (Figure 2). Such a large amount may be a real challenge to manage apple by-products. A solution to manage the AP is to integrate it directly into different products. For example, the addition of AP (5%, 10%, or 15%) in cake making can avoid the addition of other flavoring ingredients, as cakes prepared with AP had a pleasant fruity flavor (Sudha et al., 2007). The addition of 5 and 20% defatted apple seed cakes of three apple varieties to the total amount of wheat flour (WF) significantly increased the content of insoluble fiber and protein in the tested bread samples, mainly in a bread sample supplemented with 20% defatted apple seed cakes (Puric et al., 2020). Additionally, increasing consumer interest in fermented products has driven the emergence of a number of novel foods, including by-products/waste-enriched sourdough fermentation (Ganzle and Zheng, 2019; Teleky et al., 2020b).

Lactic acid bacteria (LAB) typically dominate sourdough cultures in symbiotic combination with yeasts (De Vuyst and Neysens, 2005; Van Kerrebroeck et al., 2017; Comasio et al., 2020). Enriching the sourdough fermentation with different by-products/waste increases protein digestibility and total soluble/insoluble fiber content, reduces the glycemic index of food, and improves bioavailability of minerals (De Vuyst and Neysens, 2005; Ganzle, 2014; Montemurro et al., 2019; Gobbetti et al., 2020). Therefore, the final product quality and stability are usually variable due to the uncontrolled non-sterile fermentation conditions or the substrate used and an occasional change of environmental conditions, such as temperature and water content of fermentation. The application of defined starter cultures is one of the approaches that could improve indigenous technologies and ensure starter quality consistency (Mukisa et al., 2012; Li et al., 2016).

The present article aims to capitalize on food by-products, AP, and integrate them into a continuous flow of food biotechnological processes. This manuscript integrates the performance of traditional sourdough enriched with AP and fermented by a selective LAB and yeast (mono- and co-cultures). The use of agro-industrial by-products could ensure an additional source of income and, at the same time, contribute to reducing the problem of by-product disposal and increasing the nutritional profile of fermented food products. However, there is limited information on the potential for using this specific by-product in various fermentation processes. Nonetheless, due to the results reported in this study, it is expected that the food-processing industries will better manage their by-product and waste (e.g., the integration of by-products into food to increase nutritional value), thus avoiding an environmental problem that is continuously growing.

## Materials and Methods

### Materials

*Jonathan* apples harvested during the autumn harvest period (September–November 2020) from the Bistriţa–Năsăud area (Romania) were used to obtain fresh apple juice. A discontinuous Helmut Rink GmbH press (Amtzell, Germany) was used to obtain apple juice. The moisture content of 73.70% ± 3.9% for crude AP was reduced by drying and grinding to obtain flour with 15% ± 0.5% moisture content that could be easily integrated into a fermentation process. A commercially available WF (type 000, in conformity with Romanian ash content categorization) was used, having 11.2% protein and 15.3% ± 0.3% moisture content. Culture medium components and other reagents were of analytical grade and obtained from VWR International (Radnor, Pennsylvania, PA, United States), except casein peptone obtained from Sigma-Aldrich (Steinheim, Germany) and agar (agar plant for cell culture) obtained from AppliChem (Omaha, NE, United States).

### Analysis of Organic Acids From Apple Pomace

The identification and quantification of organic acids from AP before/after dehydration were possible with high-performance liquid chromatography (HPLC), a method developed on the Agilent 1200, equipped with solvent degasser, quaternary pumps, UV detector, column thermostat, and manual injector (Agilent Technologies, United States). Chromatograms were recorded at a wavelength of λ = 210 nm. The stock solution of organic acid standards was prepared by mixing 20 μl of oxalic acid solution 300 mg/l, tartaric acid 1,000 mg/l, malic acid 2,000 mg/l, ascorbic acid 300 mg/l, citric acid 2,000 mg/l, and fumaric acid 100 mg/l. Standard organic acids from Merck were used. The HPLC sample preparation consisted of 6 ml distilled H_2_O to 1 g of AP, vortexed for 30 s, sonicated for 15 min, and centrifuged at 8,000 rpm for 10 min at 4°C. The supernatant was filtered with a Millipore membrane filter of 0.45-μm pore size. The samples were stored at −18°C until further analysis (Calinoiu et al., 2019). Organic acids were expressed as milligrams of compounds identified per gram of crude AP or AP flour, and mean ± standard deviation values were obtained for three analyses.

### Microorganisms and Culture Conditions

The microorganisms used throughout this study were *Fructilactobacillus florum* DSM No.: 22689 (*Ff*) and *Saccharomyces cerevisiae* (*Sc*) (active dry yeast—Pakmaya^®^, Izmir, Turkey) obtained from the University of Agricultural Science and Veterinary Medicine Cluj-Napoca. The medium used for *Ff* was MRS broth (glucose, 20.0 g/l; casein peptone and tryptic digest, 10.0 g/l; meat extract, 10.0 g/l; yeast extract, 5.0 g/l; Na acetate, 5.0 g/l; K_2_HPO_4_, 2.0 g/l; (NH_4_)_3_ citrate, 2.0 g/l; Tween 80, 1.00 g/l; MgSO_4_⋅7 H_2_O, 0.2 g/l; and MnSO_4_ ⋅ H_2_O, 0.05 g/l) and an addition of 5.0 g/l fructose with a final pH of 6.2–6.5. The medium used for *Sc* was GPY (glucose, 40.0 g/l; peptone, 5.0 g/l; and yeast extract, 5.0 g/l). Reactivation of the microorganisms was performed in a 9-ml MRS medium by introducing 1 ml of *Ff* inoculum or 1 g of lyophilized yeast in GPY medium. The incubation period was performed at 30°C for 24 h for both microorganisms. The propagation took place in MRS/GPY broth by inoculation of activated LAB or yeast (10 ml) with 90 ml of fresh medium and then incubated again for 24 h. *Lf* concentration of 10^8^ colony-forming units per milliliter (CFU/ml) was determined with the spectrophotometer NanoDrop 1000 (NanoDrop Technologies, Wilmington, DE, United States) through optical density measurement at 600 nm (OD600) between values 0.009 and 0.011. Yeast concentration was measured with a Thoma counting chamber (Marienfeld, Germany) under a microscope (Nikon, Japan) at a concentration of 10^6^ CFU/ml (Calinoiu et al., 2019; Teleky et al., 2020a). Also, under a microscope, possible contamination for yeast or *Ff* culture was verified.

### Sourdough Preparation and Fermentation

The sourdough preparation included a flour/water ratio of 1:1 to produce a dough yield of 300 (dough mass/flour mass 150). Dried AP was ground and added to the WF in the amount of 5 and 10%. The preparation consisted of three types of fermentation, namely, 90% WF enriched with 10% of AP flour (batch A), 95% WF enhanced with 5% AP flour (batch B), and 100% WF (batch C). Before fermentation, the measured wheat quantities went through a sterilization process, and after the addition of 120 ml of autoclaved distilled water, the dough went through a homogenization step. In addition, 30 ml of inoculum with *Sc* or 30 ml inoculum with *Ff* was used. *Ff* + *Sc* co-culture fermentation was performed, containing 90 ml of autoclaved distilled water, 30 ml inoculum with *Sc*, and 30 ml inoculum with *Ff*. The final ratio of obtained sourdough was 1 g/ml, with a final yield of 300 ml. The fermentation of the different WF + AP flour concentrations with mono- (*Ff* or *Sc*) and *Ff* + *Sc* co-cultures was carried out separately. All fermentations were performed in triplicate, in Duran bottles connected with two Millipore membrane filter plugs of 0.45-μm pore size plugged to create aerobic conditions. Sample prelevation was taken with sterile sample spatula and weighing boats. Before inoculation in WF and the mix with WF and AP flour, the culture media were centrifuged at 7,000 rpm for 10 min at 4°C, the supernatant discarded, and the pellet suspended with saline solution (Teleky et al., 2020a). After this washing step was repeated two times, with NanoDrop, LAB concentration was measured against the blank, and the yeast was counted with a Thoma counting chamber. During the fermentation, aliquots sampled for HPLC (∼5 g), viability (1 g), and pH value (5 g) were extracted at 0, 4, 8, 10, 12, 24, 48, and 72 h to monitor the changes.

### Cell Viability and pH Measurements

*Ff* and *Sc* viability was established by diluting 1 g of the sample taken in 9 ml of sterile saline solution (0.8% NaCl), processed with the pour plate method for *Ff* and spread plate method for *Sc*, and incubated for 48–72 h at 30°C. In addition, the experiments were made in triplicate, and the samples taken for the viability of LAB were evaluated through plate counting. It was displayed in logarithmic values of colony-forming units/gram of the sample (CFU/g) (Teleky et al., 2020a). In a Petri dish, 1 ml of diluted sample and about 15 ml of warm MRS agar were poured and mixed, after which it was left to solidify. Yeast viability was developed with a spread plate method on GPY agar. On the solidified agar surface, 100 μl of diluted sample was spread evenly with a sterile glass Drigalsky spatula (Calinoiu et al., 2019). Plates for *Ff* were incubated at 30°C for 48 h and for yeast at 30°C for 48–72 h. Microscopic examination was also used to investigate yeast or LAB cells as a second control check for possible contamination with different types of microorganisms. The pH value measurement in the experiments was determined with a digital pH meter (inoLab 7110, Germany) at a temperature of 22°C through dissolving 5.0 g of sample in 45 ml of distilled water, homogenized continuously with a magnetic stirrer (Laura et al., 2020).

### Secondary Metabolite Analysis by High-Performance Liquid Chromatography *HPLC*-Refractive Index Detector

After fermentation and extraction, the quantification of organic acids and secondary metabolites was possible with the help of HPLC (HPLC-Agilent 1200 Series, Santa Clara, CA, United States) equipped with a quaternary pump, solvent degasser, and manual injector coupled with refractive index detector (RID) (Agilent Technologies, CA, United States). The compounds were separated on a Polaris Hi-Plex H column, 300 × 7.7 mm (Agilent Technologies, CA, United States), using 5 mM H_2_SO_4_ mobile phase with a flow rate of 0.6 ml/min, column temperature of 80°C, and RID temperature of 35°C. Elution of the compounds was done for 25 min. Data acquisition and interpretation of results were made using the OpenLab software—ChemStation (Agilent Technologies, CA, United States). The identification of the compounds in the analyzed samples was achieved by comparing the retention times with standard compounds. The compounds evaluated during fermentation were as follows: glucose; fructose; maltose; citric acid; lactic acid; acetic acid; glycerol; 1,3-propanediol; ethanol; and two popular polyols, mannitol and erythritol. The HPLC sample preparation consisted of 2 ml distilled H_2_O to 1 g of sample, vortexed for 30 s, sonicated for 15 min, and centrifuged at 8,000 rpm for 10 min at 4°C. The supernatant was filtered with a Millipore membrane filter of 0.45-μm pore size. The experiments were made in triplicate, and the samples taken were stored at −18°C until further analysis (Teleky et al., 2020b).

### Statistical Analysis

Statistical analysis was performed with IBM SPSS Statistics 19. All tests/experiments were conducted in triplicate, and the results were expressed as the means ± standard deviation (SD). Data normality was studied using the Shapiro–Wilk test (Royston, 1992; Chen et al., 2015). One-way ANOVA with *post hoc* Tukey honestly significant difference (HSD) was used to determine if there were significant differences between batch A, batch B, and batch C for each acid, polyol, and sugar (Krzywinski and Altman, 2014). If for the value of *F* a *p* < 0.05 was obtained, the calculations were continued, and the significance of the differences was obtained for groups of two by two substrates. Scheffé, Bonferroni, and Holm *post hoc* tests were also applied to consolidate the results. In most cases, the same meaning was obtained as in the Tukey test. At each time interval, mean ± SD (*n* = 3) was passed because the data are parametric (normal). At each time interval passed, Tukey HSD *p*-value and Tukey HSD inference were as follows: on the first column, batch A vs. batch B; on the second column, batch B vs. batch C; on the third column, batch A vs. batch C. The symbols used were as follows: ^**^p < 0.01, *p < 0.05, and ^NS^*p* > 0.05.

## Results and Discussion

### Analysis of Organic Acids From Crude and Apple Pomace Flour

By-product management is challenging in order to use them efficiently, considering their necessity to be dried quickly before damage (Senevirathne et al., 2009). In this study, the drying with hot air stream was applied, which can be used economically on a commercial scale to transform the by-products into dried form. Also, the dried substrate takes up less space and is easier to store in the long term. From 271 kg of apple, 58.24 kg of AP (peel, seeds, and stem) resulted. As such, 21.49% AP was obtained, in the same range as other different publications (Giovanetti Canteri et al., 2012; Candrawinata et al., 2013; Gunes et al., 2019). After this step, the moisture content obtained for AP (73.70% ± 3.9%) is in the same range as that in the literature, which typically contains 66.4%–78.2% moisture (Shalini and Gupta, 2010; Lobo and Dorta, 2019). After drying, the substrate was ground to obtain flour with 15% ± 0.5% moisture that could be easily integrated into the fermentation process. AP flour, together with WF, was sterilized and used as a substrate to form a sourdough.

**FIGURE 3:**
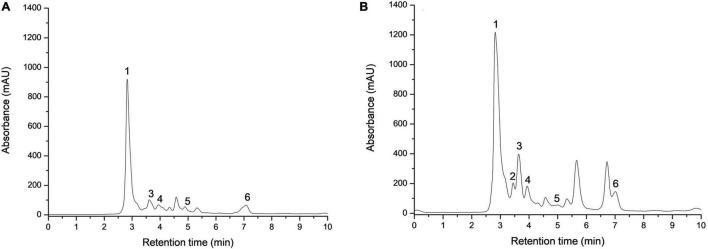
Identification of individual organic acids recovered from crude **(A)** and dried **(B)** apple pomace. 1—oxalic acid, 2—tartaric acid, 3—malic acid, 4—ascorbic acid, 5—citric acid, and 6—fumaric acid.

Apple pomace was analyzed regarding organic acids, and the extraction was made with distilled water from crude and dried AP flour. Individual organic acids of the samples were identified by HPLC. Two of the most representative chromatograms are shown in Figure 3. Data are normally distributed except for malic acid in AP after drying (0.036 < 0.05). Table 1, with statistical descriptors, describes the mean ± standard deviation (*n* = 3) in the case of normally distributed data, while for the data that were not normally distributed, the median (interquartile interval: Q1–Q3) was used.

**TABLE 1:** Organic acids from crude apple pomace and flour apple pomace.

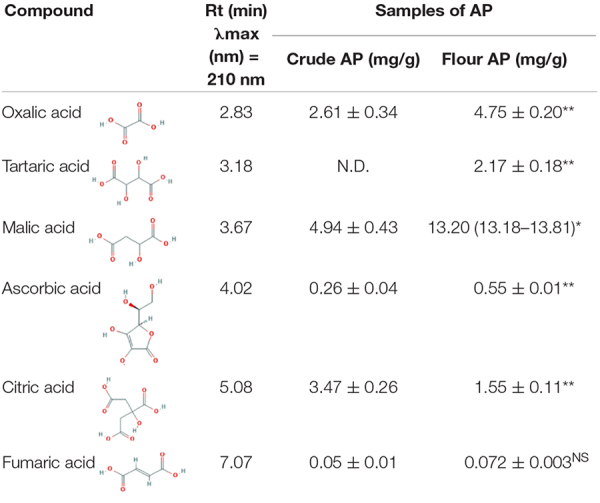

The value is expressed as milligrams of compounds identified per gram of sample (gram crude AP or gram of flour apple pomace). Mean ± standard deviation (n = 3) was added for normally distributed data, and for the data that are not normally distributed, the median (interquartile interval: Q1–Q3) was used. For the normally distributed data (parametric), the independent sample T-test was used. For malic acid after drying, considering that the data are not normally distributed (non-parametric), the Mann–Whitney test was used (**p < 0.01, *p < 0.05,

*^NS^insignificant). AP, apple pomace; N.D., not detected.*

In crude AP, 2.61 ± 0.34 mg/g oxalic acid was identified, while for dried AP flour, an increase to 4.75 ± 0.20 mg/g resulted. Tartaric acid was detected after drying with a value of 2.17 ± 0.18 mg/g. In addition, the maximal value of citric acid identified in crude AP was 3.47 ± 0.26 mg/g, while the maximal value of malic acid determined in dried AP was 13.20 mg/g. Ascorbic acid and fumaric acid were identified in a quantity of 0.26 ± 0.04 and 0.05 ± 0.01 in crude AP and in dried AP flour, respectively, and the quantity increased to 0.55 ± 0.01 and 0.072 ± 0.003, respectively.

In general, the drying process can affect a part of organic acid concentration. Apart from citric acid, higher amounts of organic acids were obtained after drying. These results are expected because dried AP samples contain less water, which means the organic acids are more concentrated in the dried matrix. However, comparing the organic acid concentration and possible losses during the drying process, we observed a relative decrease indicated as percentage, especially for citric acid with a value of −85.14%, fumaric acid with −56.67%, oxalic acid with −43.76%, ascorbic acid with −33.69%, and malic acid with −14.68%. These results showed that although hot air drying is cheap and easy to use on a large scale, the detected organic compounds were significantly degraded. Other drying alternatives for AP, but with lower direct effects in compound degradation, may be represented by lyophilization, considered for future analyses. In addition, future work could also be carried out to investigate AP degradation with microorganisms already existing in the substrate, which depreciates the substrate significantly during the transportation and storage process.

Analysis of organic acids can be a simple method for evaluating the substrate. In general, the main acids are synthesized and degraded by various metabolic pathways; for example, L-malic acid is synthesized in fruits by carboxylation of phosphoenolpyruvate in the cytosol, which originates oxaloacetate, and through the cytosolic NAD-dependent malate dehydrogenase reduction to malate. The reversibility of this reaction suggests that cytosolic MDH and the enzyme NADP-malic acid are involved in both the synthesis of malate and the degradation during ripening of several fruit species, a process apparently stimulated by high temperatures (Mendes Ferreira and Mendes-Faia, 2020). In general, total acidity tends to decrease the sugar content; for example, in ripe grapes, acid levels tend to be lower in the warmer climate than in colder ones, with tartaric acid being the predominant acid due to its higher stability at higher temperatures. In addition, during fermentation with microorganisms already existing in the substrate, the acids undergo relevant changes directly or indirectly due to metabolic activity; this drastically leads to the devaluation of the substrate.

Generally, oxalic acid in food can chelate minerals and inhibit their absorption. However, oxalic acid is commonly found in plants such as spinach, rhubarb, beetroot, banana, and apple (Magnuson and Lasure, 2004; Betiku et al., 2016). Nonetheless, AP may be a cheap and important source of oxalic acid from a “green” source (Flores et al., 2012).

The addition of tartaric acid, citric acid, or malic acid to grape-based beverages has become a common industrial practice due to the stabilizing effect of these organic acids and the resulting ability to increase the product’s shelf life (Gurtler and Mai, 2014). Tartaric acid has a stronger and sharper taste than citric acid. Although it is famous for its natural appearance in grapes, it also occurs in apples, cherries, papaya, peach, pear, pineapple, strawberry, mango, and citrus. In addition, L-tartaric acid degradation is associated with species of basidiomycete affinity (Senevirathne et al., 2009; Flores et al., 2012). Unlike tartaric acid, malic acid is easily degraded by most microorganisms; a low concentration of malic acid indicates contamination in the substrate (Senevirathne et al., 2009). In particular, citric acid is highly favored by the food industry because of its light fruity taste, solubility, low cost, and abundant supply (Gurtler and Mai, 2014). Also, citric acid contributes to the acidity of AP (Bhushan et al., 2008; Vendruscolo et al., 2008; Senevirathne et al., 2009; Flores et al., 2012; Scherer et al., 2012).

### Viable Cell Count and pH Measurements During Sourdough Fermentation

**FIGURE 4:**
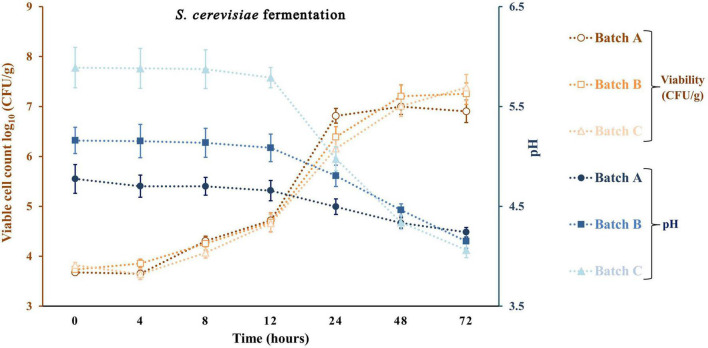
Viable cell count and pH values for the sourdough fermentation using *Saccharomyces cerevisiae*. Three types of fermentation with 90% wheat flour (WF) and 10% of apple pomace (AP) addition (batch A), 95% WF with 5% AP (batch B), and 100% WF (batch C) were prepared. Values for yeast viable cell growth and pH are displayed as mean values ± SD, log_10_ CFU/g, *n* = 3 (CFU/g, colony-forming units/gram of the sample).

**FIGURE 5:**
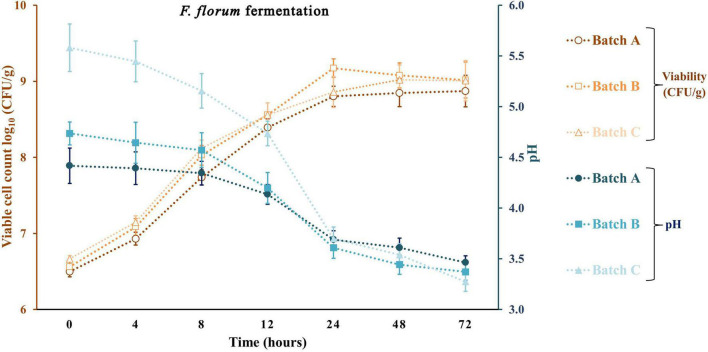
Viable cell count and pH values for the sourdough fermentation using *Fructilactobacillus florum*. Three types of fermentation with 90% WF and 10% of AP addition (batch A), 95% WF with 5% AP (batch B), and 100% WF (batch C) were prepared. Values for lactic acid bacteria (LAB) viable cell growth and pH are displayed as mean values ± SD, log_10_ CFU/g, *n* = 3 (CFU/g, colony-forming units/gram of the sample).

**FIGURE 6:**
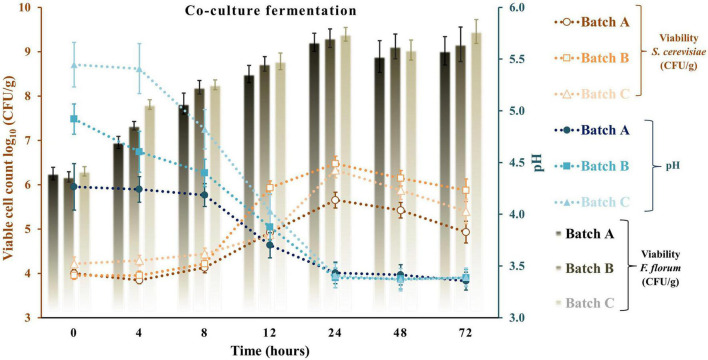
Viable cell count and pH values for the sourdough fermentation using co-culture fermentation of *Saccharomyces cerevisiae* + *Fructilactobacillus florum*. Three types of fermentation with 90% WF and 10% of AP addition (batch A), 95% WF with 5% AP (batch B), and 100% WF (batch C) were prepared. Values for LAB or yeast cell count and pH are displayed as mean values ± SD, log_10_ CFU/g, *n* = 3 (CFU/g, colony-forming units/gram of the sample).

Apple pomace by-products at 5 and 10% were the ingredients used to fortify a traditional sourdough fermentation with a selective LAB and yeast. With the same approach, previous studies did not exceed the 10% fortification with fruit by-products. Typically, at values above 10%, a significant loss of bread acceptability was observed (Gunes et al., 2019; Puric et al., 2020). Figures 4–6 represent all the three studied batches: batch A: 90% WF enriched with 10% of AP flour; batch B: 95% WF enriched with 5% AP flour; and batch C: 100% WF. According to the figures, which represent cell growth during 72 h of fermentation using *Sc* (Figure 4), *Ff* (Figure 5), and *Sc* + *Ff* co-culture (Figure 6), each microorganism’s growth increased with an increase in the time of fermentation. Fermentations reached the number of *Ff* viable cells around 8.9 and 9.2 log_10_ CFU/g for all fermentation batches. This number of cells was reached during the first 24 h of fermentation, and a constant decrease of pH was observed during this time. These results indicate that the fermentation finalization of sourdough is completed in the first 24 h. In addition, in the first 24 h, the analysis of the viable cell count of microorganisms showed a high increase, especially for *Ff* single fermentation and in *Ff* + *Sc* co-cultures with a final concentration of over 9.0 log_10_ CFU/g.

*Ff*’s highest viable cell count was with substrate 95% WF and 5% AP (9.1 log_10_ CFU/g). In the *Ff* + *Sc* co-culture fermentation, *Ff*’s highest growth was identified in the fermentation having 100% WF, the substrate showing a fast growth when compared to *Ff* monoculture fermentation where a constant growth was shown during the experiment. Simultaneously, *Sc* in *Ff* + *Sc* co-culture showed slower growth, but the final concentration was a maximum of 6.5 log_10_ CFU/g in the batch B fermentation. No adverse effects on LAB viability were observed in the presence of *Sc*. At the same time, the *Sc* in the presence of *Ff* showed a slight decrease in the viability for all three substrates, and these findings were also observed in model media culture. The minimum viable cells of 5.6 log_10_ CFU/g were visible in batch C. Oppositely, AP flour + WF had a positive effect, especially in fermentations with 95% WF and 5% AP flour, predominantly in *Ff* + *Sc* co-culture fermentation. Overall, comparing the results with similar studies (Hashemi et al., 2019; Teleky et al., 2020b), LAB showed the maximum number of surviving cells during 24 h of fermentation; in this study, the maximum number of surviving cells was also observed after 24 h. In general, yeast cell growth can be partially inhibited in the dough with mixed LAB cultures due to the rapid decrease of pH and the accumulation of lactic or acetic acid in an undissociated, lipophilic, and membrane-diffusible form, combined with the negative effects of ethanol on growth (Paramithiotis et al., 2006; Paucean et al., 2013). This inhibition is also observable from the viability of fermentation with *Ff* + *Sc* co-culture. An explanation may be that although the presence of acetic acid in co-culture was lower than that in pure fermentation with *Sc*, the presence of lactic acid was relatively similar for pure *Ff* culture and co-culture. Besides, another explanation may be the ethanol presence in higher amounts in the co-culture compared to the pure culture, which inhibits acetic acid production. In *Ff* + *Sc* co-culture, *Sc* reached a maximum of 6.4 log_10_ CFU/g compared with *Sc* monoculture fermentation, where the viable cells got the maximum value of 7.4 log_10_ CFU/g.

The production of lactate and lactic acid by LAB during fermentation leads to a reduction in the pH. The continued metabolic activity of lactobacilli causes a further decrease in pH values until a critical limit is reached, especially for yeasts (Hashemi et al., 2019). After 72 h of fermentation, the lowest pH value was obtained in fermentation with *Ff* and 100% WF. Comparing the value obtained in batch C and batch A with 10% AP, batch A shows a constant and linear decrease in pH throughout the fermentation. A similar decline in pH was reported for different types of sourdoughs in various fermentation conditions (Kockova et al., 2013; Hashemi et al., 2019; Teleky et al., 2020b). A similar reduction in pH could be observed in a study on different carbohydrates with starting values of around 6.4 and final values between 3.1 and 3.5 (Hashemi et al., 2019). The minimum value in this study was between 4.24 ± 0.04 for *Sc* fermentation and 3.27 ± 0.10 for *Ff* fermentation. Many studies show that the pH can be lowered by reaching final values of 4.57 with *Sc* fermentations in substrate containing only glucose as carbohydrate or different sourdough fermentations (Paucean et al., 2013). On the other hand, LAB growth as monocultures resulted in a radical pH decrease from range 5.58 ± 0.23 to 3.28 ± 0.10 in batch C, slightly lower for batch B from 4.74 ± 0.11 to 3.37 ± 0.08 and lower in batch A from 4.42 ± 0.17 to 3.46 ± 0.07. The *Ff* + *Sc* co-culture did not affect the pH; the final pH value ranged from 3.35 ± 0.09 to 3.39 ± 0.09. In addition, crude AP had a pH value of 4.03 ± 0.07, which means an addition of AP in a fermentation process can decrease the initial pH value (Cantatore et al., 2019).

### Organic Acids and Secondary Metabolite Analysis During Sourdough Fermentation

The compounds evaluated during fermentation were glucose; fructose; maltose; citric acid; lactic acid; acetic acid; glycerol; 1,3-propanediol; ethanol; and two popular polyols, mannitol and erythritol. Tables 2–4 show the results obtained during the fermentation process. AP is rich in fermentable sugars, like fructose (19.2%) and sucrose (1.0%) (Magyar et al., 2016). In general, fructose was from residual juice left behind in AP after pressing and can be easily metabolized by *Fructilactobacillus* spp. Subsequent water evaporation during drying deposited soluble fructose on the pomace surface. In addition, sucrose is a major AP component. However, sucrose rapidly breaks down into glucose and fructose monosaccharides. Therefore, the received AP sucrose content is difficult to measure accurately, and measuring glucose and fructose equivalents is more accurate. In all the fermentation processes, fructose and glucose have maximum values for time 0 and after 4 h, with values ranging between 7.46 ± 0.60 and 12.55 ± 0.38 g/l. Overall, the pomace contains several carbohydrates and organic acids that are native and may be an important source of fermentable substrate for bacteria and yeasts. Our results show that enriched WF with AP flour increases the substrate substantially with fructose, glucose, and maltose. The consumption of sugar indicates the capability of cultures during fermentation and substrate transformation of yeasts or bacteria (Magyar et al., 2016; Wang et al., 2020).

**TABLE 2:** The amount of carbohydrates, organic acids, and alcohols for *Sc* fermentation.

	Time	Batch A	Batch B	Batch C
Fructose	0	8.85 ± 0.08**	5.64 ± 0.06**	0.73 ± 0.02**
	4	9.15 ± 0.08**	5.34 ± 0.09**	0.89 ± 0.09**
	8	9.69 ± 0.06**	5.39 ± 0.08**	0.84 ± 0.05**
	12	8.76 ± 0.10**	4.86 ± 0.09**	0.72 ± 0.02**
	24	0.81 ± 0.02**	0.28 ± 0.06**	N.D.
	48	0.18 ± 0.03**	N.D.	N.D.
	72	0.05 ± 0.01**	N.D.	N.D.
Glucose	0	3.27 ± 0.17**	2.26 ± 0.06**	0.46 ± 0.07**
	4	3.33 ± 0.06**	2.06 ± 0.06**	0.54 ± 0.04**
	8	3.44 ± 0.06**	1.94 ± 0.05**	0.43 ± 0.02**
	12	2.86 ± 0.06**	1.19 ± 0.07**	0.22 ± 0.03**
	24–72	N.D.	N.D.	N.D.
Maltose	0	1.25 ± 0.03**	0.85 ± 0.04*	0.67 ± 0.07**
	4	1.48 ± 0.09**	1.16 ± 0.05**	1.09 ± 0.07^NS^(0.574)
	8	1.42 ± 0.06^NS^(0.326)	1.36 ± 0.04**	1.16 ± 0.03**
	12	1.53 ± 0.10^NS^(0.626)	1.33 ± 0.06^NS^(0.899)	1.48 ± 0.43^NS^(0.756)
	24	0.32 ± 0.01**	0.23 ± 0.02**	0.20 ± 0.01^NS^(0.110)
	48	0.17 ± 0.01**	0.10 ± 0.01**	0.11 ± 0.01^NS^(0.688)
	72	0.09 ± 0.01^NS^(0.783)	0.095 ± 0.002*	0.13 ± 0.01*
Citric acid	0	0.09 ± 0.01**	0.052 ± 0.001**	0.038 ± 0.001**
	4	0.093 ± 0.003**	0.056 ± 0.003**	0.049 ± 0.002*
	8	0.09 ± 0.01**	0.053 ± 0.008**	0.043 ± 0.006^NS^(0.408)
	12	0.09 ± 0.01*	0.054 ± 0.002**	0.052 ± 0.007^NS^(0.899)
	24	0.09 ± 0.01**	0.046 ± 0.001**	0.044 ± 0.002^NS^(0.899)
	48	0.052 ± 0.002*	0.032 ± 0.002^NS^(0.055)	0.032 ± 0.001^NS^(0.867)
	72	0.05 ± 0.01*	0.021 ± 0.001*	0.037 ± 0.001^NS^(0.782)
Lactic acid	0–12	N.D.	N.D.	N.D.
	24	0.063 ± 0.003**	0.101 ± 0.002**	0.131 ± 0.003*
	48	0.20 ± 0.01**	0.27 ± 0.02**	0.14 ± 0.01**
	72	0.28 ± 0.01**	0.37 ± 0.02^NS^(0.090)	0.24 ± 0.01**
Acetic acid	0–12	N.D.	N.D.	N.D.
	24	N.D.	0.08 ± 0.01**	0.29 ± 0.02**
	48	0.12 ± 0.02**	0.30 ± 0.02**	0.30 ± 0.01^NS^(0.852)
	72	0.23 ± 0.01^NS^(0.899)	0.23 ± 0.01*	0.28 ± 0.02*
Mannitol	0–12	N.D.	N.D.	N.D.
	24	0.161 ± 0.001**	0.084 ± 0.001**	N.D.
	48	0.26 ± 0.01**	0.18 ± 0.01**	N.D.
	72	0.37 ± 0.04**	0.183 ± 0.001**	N.D.
Erythritol	0–12	N.D.	N.D.	N.D.
	24	0.05 ± 0.01^NS^(0.483)	0.04 ± 0.01^NS^(0.702)	0.062 ± 0.001^NS^(0.184)
	48	0.095 ± 0.003*	0.06 ± 0.01^NS^(0.265)	0.071 ± 0.02^NS^(0.350)
	72	0.10 ± 0.01**	0.07 ± 0.01^NS^(0.237)	0.092 ± 0.004^NS^(0.068)
Glycerol	0	0.047 ± 0.002^NS^(0.324)	0.034 ± 0.001**	N.D.
	4	0.06 ± 0.05^NS^(0.769)	0.04 ± 0.03^NS^(0.198)	N.D.
	8	0.06 ± 0.06^NS^(0.899)	0.05 ± 0.04^NS^(0.281)	N.D.
	12	0.064 ± 0.001^NS^(0.651)	0.053 ± 0.003**	0.019 ± 0.001**
	24	0.22 ± 0.01**	0.12 ± 0.01**	0.041 ± 0.002**
	48	0.21 ± 0.01**	0.142 ± 0.002**	0.048 ± 0.006**
	72	0.22 ± 0.01**	0.13 ± 0.01**	0.014 ± 0.001**
Ethanol	0	0.85 ± 0.07**	1.26 ± 0.06*	0.67 ± 0.05**
	4	1.17 ± 0.07*	1.47 ± 0.11^NS^(0.510)	1.08 ± 0.09**
	8	1.37 ± 0.03^NS^(0.686)	1.45 ± 0.18^NS^(0.149)	1.17 ± 0.06^NS^(0.051)
	12	1.34 ± 0.04*	1.56 ± 0.09^NS^(0.127)	1.22 ± 0.05**
	24	0.23 ± 0.01**	0.32 ± 0.01^NS^(0.136)	0.20 ± 0.02**
	48	0.17 ± 0.01**	0.10 ± 0.01*	0.13 ± 0.01^NS^(0.060)
	72	0.09 ± 0.01^NS^(0.625)	0.10 ± 0.01*	0.13 ± 0.01*
1,3-propanediol	0–72	N.D.	N.D.	N.D.

*The carbohydrates, organic acids, and alcohols were expressed as grams of compounds identified per liter of extract (g/l). The experiments were made in triplicate, and the values represent the average and standard deviation of them. Data normality was studied using the Shapiro–Wilk test. For values, p > 0.05, the data are normally distributed, and in the descriptive statistics table, we passed mean ± SD (n = 3). One-way ANOVA with post hoc Tukey HSD was used to determine if there were significant differences between batch A, batch B, and batch C for each compound. The p < 0.05 value corresponding to the F-statistic of one-way ANOVA was obtained, the calculations were continued, and the significance of the differences for groups of two by two substrates was obtained. In brackets, Tukey HSD p-value and Tukey HSD inference were passed as follows: on the first column, batch A vs. batch B; on the second column, batch B vs. batch C; on the third column, batch A vs. batch C.*

The symbols were used as follows: **p < 0.01, *p < 0.05, ^NS^(insignifiant) p > 0.05. N.D., not detected.

The carbohydrate consumption capability of *Ff* strains is presented in Table 3, as after 72 h, glucose decreases until 0.36 ± 0.06 g/l in batch A, 0.92 ± 0.11 g/l in batch B, and 0.11 ± 0.03 g/l in batch C. However, *Sc* consumed glucose entirely during 24 h, compared with *Ff* fermentation, where glucose is consumed gradually. In addition, fermentation with just *Ff* decreases glucose, fructose, and maltose, but not entirely. Also, Bartkiene et al. (2019) showed LAB capability, and the highest carbohydrate fermentation diversity was by *Lactobacillus coryniformis* LUHS71 and *Lactilactobacillus curvatus* LUHS51 (23 and 22 carbohydrates from 49 analyzed, respectively), and the lowest was by *Lactobacillus farraginis* LUHS206 (10 carbohydrates).

**TABLE 3:** The amount of carbohydrates, organic acids, and alcohols for *Ff* fermentation.

	TIMES	Batch A	Batch B	Batch C
Fructose	0	7.460.60**	2.560.23**	0.730.10**
	4	5.390.43**	1.650.13**	0.330.04**
	8	5.670.46**	2.770.30**	0.270.03**
	12	6.200.50**	3.810.35**	0.200.02**
	24	4.280.37^NS^(0.078)	3.660.32**	0.100.02**
	48	5.360.46**	2.820.27**	0.050.01**
	72	2.500.24**	3.610.33**	0.050.01**
Glucose	0	3.610.35**	1.200.13**	0.260.07**
	4	2.060.20**	0.730.08**	0.540.08^NS^(0.268)
	8	2.370.23**	1.230.13**	0.900.09^NS^(0.102)
	12	2.390.23**	1.380.14**	0.710.08**
	24	1.070.11^NS^(0.899)	1.090.11**	0.420.06**
	48	1.100.11^NS^(0.194)	0.940.11**	0.360.06**
	72	0.360.06**	0.920.11*	0.110.03**
Maltose	0	1.320.15**	0.640.07**	0.570.05^NS^(0.729)
	4	0.880.09**	0.530.01^NS^(0.687)	0.840.04**
	8	1.080.07^NS^(0.752)	1.050.04**	1.440.06**
	12	1.250.03**	1.490.09*	1.050.03**
	24	0.960.05**	1.780.11**	0.300.04**
	48	1.230.03**	1.370.04**	N.D.
	72	0.450.04**	1.730.08**	N.D.
Citric acid	0	0.210.04**	0.080.02**	0.0140.001*
	4	0.160.04**	0.050.01**	0.0130.001^NS^(0.189)
	8	0.130.01**	0.090.01**	N.D.
	12	0.140.04^NS^(0.417)	0.120.01**	N.D.
	24	0.020.01**	0.090.02^NS^(0.315)	N.D.
	48	0.0130.001*	0.070.02^NS^(0.542)	N.D.
	72	N.D.	0.130.02^NS^(0.899)	N.D.
Lactic acid	0–4	N.D.	N.D.	N.D.
	8	0.130.02^NS^(0.899)	0.130.03^NS^(0.283)	0.170.03^NS^(0.184)
	12	0.420.04^NS^(0.315)	0.380.03**	0.300.02*
	24	0.960.06**	0.670.05**	0.560.07^NS^(0.124)
	48	1.300.02^NS^(0.057)	1.090.07*	1.050.10^NS^(0.851)
	72	2.500.21**	1.540.11**	1.300.09^NS^(0.188)
Acetic acid	0–12	N.D.	ND.	N.D.
	24	N.D.	ND.	0.030.01**
	48	N.D.	ND.	0.040.02*
	72	N.D.	ND.	0.100.01**
Mannitol	0–72	N.D.	ND.	N.D.
Erythritol	0–72	N.D.	ND.	N.D.
Glycerol	0	0.020.01^NS^(0.174)	0.040.02^NS^(0.271)	N.D.
	4	0.020.01^NS^(0.107)	0.040.01^NS^(0.370)	0.030.01^NS^(0.606)
	8	0.030.01^NS^(0.899)	0.040.01^NS^(0.214)	0.020.01^NS^(0.154)
	12	0.040.01^NS^(0.405)	0.050.01^NS^(0.154)	0.020.01*
	24	0.040.01^NS^(0.899)	0.040.02**	N.D.
	48–72	N.D.	N.D.	N.D.
Ethanol	0–72	N.D.	N.D.	N.D.
1,3-propanediol	0–72	N.D.	N.D.	N.D.

*The carbohydrates, organic acids, and alcohols were expressed as grams of compounds identified per liter of extract (g/l). The experiments were made in triplicate, and the values represent the average and standard deviation of them. Data normality was studied using the Shapiro–Wilk test. For values, p > 0.05, data are normally distributed, and in the descriptive statistics table, we passed mean ± SD (n = 3). One-way ANOVA with post hoc Tukey HSD was used to determine if there were significant differences between batch A, batch B, and batch C for each compound. The p < 0.05 value corresponding to the F-statistic of one-way ANOVA was obtained, the calculations were continued, and the significance of the differences for groups of two by two substrates was obtained. In brackets, Tukey HSD p-value and Tukey HSD inference were passed as follows: on the first column, batch A vs. batch B; on the second column, batch B vs. batch C; on the third column, batch A vs. batch C.*

The symbols were used as follows: **p < 0.01, *p < 0.05, ^NS^(insignifiant) p > 0.05. N.D., not detected.

**TABLE 4:** The amount of carbohydrates, organic acids, and alcohols for *Ff* + *Sc* fermentation.

	TIMES	Batch A	Batch B	Batch C
Fructose	0	8.48 ± 0.25**	4.87 ± 0.21**	0.94 ± 0.06**
	4	10.40 ± 0.36**	4.73 ± 0.22**	1.00 ± 0.07**
	8	12.55 ± 0.38**	4.61 ± 0.11**	0.84 ± 0.05**
	12	12.45 ± 0.36**	4.44 ± 0.13**	0.33 ± 0.05**
	24	0.98 ± 0.09**	0.31 ± 0.02**	N.D.
	48	0.58 ± 0.10**	N.D.	N.D.
	72	0.46 ± 0.09**	N.D.	N.D.
Glucose	0	2.69 ± 0.19**	1.85 ± 0.22**	0.52 ± 0.04**
	4	3.77 ± 0.23**	1.69 ± 0.23**	0.36 ± 0.06**
	8	4.40 ± 0.27**	1.53 ± 0.15**	0.07 ± 0.02**
	12	3.75 ± 0.21**	0.95 ± 0.15**	N.D.
	24	0.23 ± 0.03**	N.D.	N.D.
	48–72	N.D.	N.D.	N.D.
Maltose	0	1.65 ± 0.20**	0.78 ± 0.09**	0.69 ± 0.07^NSx^(0.683)
	4	2.66 ± 0.28**	1.34 ± 0.14**	0.71 ± 0.07*
	8	3.64 ± 0.35**	1.23 ± 0.14**	0.47 ± 0.07*
	12	3.88 ± 0.35**	0.93 ± 0.11**	0.06 ± 0.01**
	24	0.19 ± 0.03**	0.09 ± 0.02**	N.D.
	48–72	N.D.	N.D.	N.D.
Citric acid	0	0.12 ± 0.01^NSx^(0.346)	0.10 ± 0.01^NSx^(0.899)	0.12 ± 0.01^NSx^(0.286)
	4	0.17 ± 0.02*	0.12 ± 0.01^NSx^(0.899)	0.17 ± 0.02*
	8	0.20 ± 0.02*	0.13 ± 0.02^NSx^(0.503)	0.18 ± 0.04^NSx^(0.158)
	12	0.23 ± 0.04*	0.13 ± 0.03^NSx^(0.327)	0.18 ± 0.05^NSx^(0.346)
	24	0.26 ± 0.01*	0.20 ± 0.02**	0.18 ± 0.02^NSx^(0.608)
	48	0.24 ± 0.03^NSx^(0.451)	0.21 ± 0.02^NSx^(0.564)	0.21 ± 0.02^NSx^(0.899)
	72	0.22 ± 0.02^NSx^(0.115)	0.18 ± 0.02**	0.10 ± 0.01**
Lactic acid	0	N.D.	N.D.	N.D.
	4	N.D.	0.05 ± 0.04^NSx^(0.899)	N.D.
	8	0.17 ± 0.02*	0.28 ± 0.03**	0.34 ± 0.05^NSx^(0.218)
	12	0.51 ± 0.02^NSx^(0.247)	0.58 ± 0.03**	0.74 ± 0.06**
	24	0.86 ± 0.06*	1.45 ± 0.10^NSx^(0.899)	0.85 ± 0.06**
	48	1.45 ± 0.09^NSx^(0.051)	1.62 ± 0.05^NSx^(0.696)	1.41 ± 0.05*
	72	1.56 ± 0.07^NSx^(0.781)	1.51 ± 0.08^NSx^(0.102)	1.38 ± 0.11^NSx^(0.236)
Acetic acid	0–24	N.D.	N.D.	N.D.
	48	0.07 ± 0.01^NSx^(0.270)	0.09 ± 0.02**	N.D.
	72	0.10 ± 0.01**	0.26 ± 0.03^NSx^(0.283)	0.07 ± 0.02**
Mannitol	0–12	N.D.	N.D.	N.D.
	24	0.31 ± 0.03**	0.18 ± 0.02**	N.D.
	48	0.42 ± 0.03**	0.14 ± 0.02**	N.D.
	72	0.39 ± 0.04**	0.13 ± 0.02**	N.D.
Erythritol	0–12	N.D.	N.D.	N.D.
	24	0.03 ± 0.01^NSx^(0.739)	0.04 ± 0.02^NSx^(0.481)	0.05 ± 0.02^NSx^(0.200)
	48	0.05 ± 0.02**	0.02 ± 0.01^NSx^(0.124)	0.04 ± 0.02^NSx^(0.138)
	72	0.05 ± 0.02*	0.03 ± 0.01**	0.014 ± 0.001^NSx^(0.380)
Glycerol	0	0.04 ± 0.02**	N.D.	N.D.
	4	0.06 ± 0.02*	0.03 ± 0.01**	N.D.
	8	0.09 ± 0.01^NSx^(0.166)	0.49 ± 0.40^NSx^(0.871)	N.D.
	12	0.39 ± 0.51^NSx^(0.883)	0.28 ± 0.05^NSx^(0.308)	N.D.
	24	0.22 ± 0.02*	0.17 ± 0.02**	0.03 ± 0.01**
	48	0.30 ± 0.03**	0.14 ± 0.02**	0.04 ± 0.01**
	72	0.20 ± 0.15^NSx^(0.572)	0.12 ± 0.01^NSx^(0.267)	0.07 ± 0.01^NSx^(0.768)
Ethanol	0–4	N.D.	N.D.	N.D.
	8	N.D.	N.D.	0.69 ± 0.06**
	12	0.48 ± 0.03**	0.25 ± 0.04**	1.86 ± 0.08*
	24	4.53 ± 0.15^NS^(0.053)	4.24 ± 0.12**	2.28 ± 0.08*
	48	6.18 ± 0.22**	3.65 ± 0.27**	1.71 ± 0.08*
	72	5.59 ± 0.26**	2.88 ± 0.17**	1.15 ± 0.05*
1,3-propanediol	0–72	N.D.	N.D.	N.D.

*The carbohydrates, organic acids, and alcohols were expressed as grams of compounds identified per liter of extract (g/l). The experiments were made in triplicate, and the values represent the average and standard deviation of them. Data normality was studied using the Shapiro–Wilk test. For values, p > 0.05, data are normally distributed, and in the descriptive statistics table, we passed mean ± SD (n = 3). One-way ANOVA with post hoc Tukey HSD was used to determine if there were significant differences between batch A, batch B, and batch C for each compound. The p < 0.05 value corresponding to the F-statistic of one-way ANOVA was obtained, the calculations were continued, and the significance of the differences for groups of two by two substrates was obtained. In brackets, Tukey HSD p-value and Tukey HSD inference were passed as follows: on the first column, batch A vs. batch B; on the second column, batch B vs. batch C; on the third column, batch A vs. batch C.*

The symbols were used as follows: **p < 0.01, *p < 0.05, ^NS^(insignifiant) p > 0.05. N.D., not detected.

Mannitol production by microorganisms has been extensively studied using batch fermentation. Nevertheless, the batch mode suffers from a relatively low mannitol concentration and productivity due to inhibition of substrate with mannitol (Martau et al., 2020). Mannitol results from fructose utilization as an electron acceptor and its concomitant reduction, and it is a characteristic of some heterofermentative sourdough LAB. For example, *Fructilactobacillus* (*F.*) *sanfranciscensis* (formerly *Lactobacillus sanfranciscensis*) and *Levilactobacillus brevis* strains were able to utilize fructose in such a manner, resulting in the production of 0.003 mmol/g sourdough mannitol when grown as a monoculture. It became known that yeast co-culture resulted in a significant increase in mannitol production of 0.03 mmol/g sourdough and 0.05 mmol/g sourdough when the yeast was co-cultured with *F. sanfranciscensis* and *L. brevis*, respectively (Paramithiotis et al., 2006). In our fermentation, during 72 h, *Ff* does not produce erythritol and mannitol under aerobic conditions. Similarly, *Sc* produced mannitol in batch A and batch B after 24 h, and the maximum values were 0.37 ± 0.04 and 0.183 ± 0.001 g/l, respectively, after 72 h. Mannitol was not detected in any of the batch C fermentation processes, meaning that the addition of AP in a fermentation process with *Sc* induces the mannitol production. *Ff* + *Sc* co-culture fermentation also increases mannitol production with a maximum value of 0.42 ± 0.03 g/l but decreases erythritol production with a maximum value of 0.05 ± 0.02 g/l. Mannitol, erythritol, ethanol, and 1,3-propanediol were not detected in *Ff* fermentation.

Lactic acid was identified in the range of 8–24 h of fermentation. The maximum value was identified after 48 and 72 h, especially in batch A, *Ff* fermentation, which means AP has a positive influence on lactic acid production. In addition, *Ff* + *Sc* co-culture fermentation reduced lactic acid production, and a cause of this may reside in carbohydrate availability (Li et al., 2021).

Acetic acid is another end product of heterofermentative metabolism. The monoculture of all heterofermentative LAB studied resulted in the production of non-traceable amounts of acetic acid. In contrast, according to a previous study, the co-culture with yeast resulted in the production of 0.01 mmol/g sourdough by *F. sanfranciscensis* and *L. brevis* and 0.02 mmol/g sourdough by *Weissella cibaria* (Paramithiotis et al., 2006). In our case, the maximum value obtained was 0.30 ± 0.02 g/l in *Sc* fermentation with 95% WF and 5% AP.

Glycerol is a by-product of alcoholic fermentation of the yeast. In a previous study, when the yeast was grown as a monoculture, 0.03 mmol/g sourdough was produced (Paramithiotis et al., 2006). Glycerol production seemed to be positively affected by yeast co-culture with *F. sanfranciscensis* or *L. brevis* and negatively affected by the co-culture with *Pediococcus pentosaceus* or *Enterococcus faecium*, while no effect was observed when yeast was grown with *W. cibaria* or *Lactobacillus paralimentarius* (Paramithiotis et al., 2006). In our experiment, *Sc* fermentation generated a maximum glycerol value of 0.22 ± 0.01 g/l in batch A and 0.142 ± 0.002 g/l in batch B compared to batch C, where the maximum glycerol value was 0.048 ± 0.006. In addition, co-culture fermentation doubled glycerol production in batch A to a maximum value of 0.39 ± 0.51 g/l, tripled in batch B toward 0.49 ± 0.40 g/l, and almost doubled to 0.07 ± 0.01 g/l in batch C. *Ff* maintained a constant value during the fermentation process between 0.02 ± 0.01 and 0.05 ± 0.01 g/l.

*Sc* is the most popular yeast in the production of ethanol due to its wide tolerance of pH. Yeast’s ability to catabolize six-carbon molecules is the bedrock to bioethanol production without proceeding to the final product of oxidation, which is CO_2_ (Chen et al., 2008). Observably, ethanol presence was detected in all fermentations with *Sc*. The maximum value of ethanol was identified in *Sc* + *Ff* co-culture fermentation, batch A 6.18 ± 0.22; also, *Sc* fermentation has 70% lower ethanol compared to *Ff* + *Sc* co-culture fermentation. However, *Sc* + *Ff* has a positive influence on ethanol production, especially in sourdough fermentation with AP.

Usually, bread properties are greatly affected by sourdough stability; microbial strain stability is very important (Alfonzo et al., 2016). LAB stability in a sourdough ecosystem is influenced by various factors, including specific metabolic adaptations to the sourdough ecosystem and metabolic interactions. Conversely, using a sterile WF, the properties of sourdough can be controlled by the added microorganism mix. All of the strains showed high fermentation activity on fructose, glucose, and maltose, the main soluble carbohydrates of sourdough. Regarding the results obtained, the *Ff* indicating a high tolerance to acidic conditions and overall carbohydrate metabolism can be recommended for sourdough bread production (Bartkiene et al., 2019). Furthermore, this study is an explorative study that needs validation in a real bakery together with a microbiota monitoring and therefore represents the perspectives of our future work experiments. These laboratory-produced sourdough analyses should focus on microbiota development in flours, starting with flour as the only non-sterile ingredient (De Vuyst et al., 2014). However, *de novo* preparation of sourdoughs in bakeries may involve using an inoculum to accelerate the establishment of suitable fermentation microbiota (Ripari et al., 2016). Also, in some cases, competition between the spontaneously growing microbiota and the added sourdough starter culture may lead to the dominance of autochthonous LAB species or strains and hence eliminate the added started culture. This is possibly due to the lack of adaptation of the starter culture to the environmental conditions of the particular sourdough ecosystem, and this feature needs to be evaluated in future analyses (Siragusa et al., 2009; Minervini et al., 2010; Moroni et al., 2010).

Overall, the processing industry releases a massive amount of by-products, usually in short harvest periods, and thus, storage and damage problems of these products can be encountered. Therefore, it is necessary to dry these by-products quickly before damage in order to use them efficiently (Senevirathne et al., 2009; Nemes et al., 2020). We believe that our results underline the need for a comprehensive system for AP’s valorization, reducing environmental pollution, and AP’s capitalization by integrating it into sourdough processes. By-product management is challenging in eliminating huge amounts of by-products, as they have a high biological demand for oxygen.

Apple pomace by-products are an important source of organic acids, a source of fiber, or a substrate in biotechnological processes (Vendruscolo et al., 2008). Biotechnological applications of the AP are interesting from the viewpoint of a low-cost substrate and solving problems related to AP by-product disposal, a pollution source that has been gaining much attention in apple-producing areas. The food fortification with fruit by-products (apple, banana, grapes, citrus, and berries) has already been addressed, the interest being noticed in numerous researches. The future perspective needs to be focused on fruit by-products that can delay the drying of bread (Ganzle and Zheng, 2019; Gunes et al., 2019). In principle, ripe products are basic foods suitable for enrichment. The use of highly processed ingredients and industrialized working methods has thus caused a reduction in sensory attributes.

The analysis of Tables 2–4 shows that one of the most important contributions of AP to the sourdough is the fermentable sugars (in wheat flour doughs, these sugars are limited). These sugars extended the fermentation time by favoring mainly lactic fermentation in co-cultures. Also, AP by-products are certainly an important source of dietary fiber. Dietary fiber slows down many processes associated with the digestion of glycemic carbohydrates, such as gastric emptying, small intestinal transit, and transport from the lumen to the mucosal surface. Therefore, dietary fiber reduces the risk of type 2 diabetes and improves insulin/glucose metabolism. The additional characteristics of WF fortified with AP are also noticeable by increasing the shelf life. Apple pomace, as previously reported, contains inhibitory compounds, such as specific polyphenols like chlorogenic acid, epicatechin, and phloridzin, which have been shown to have antimicrobial activities against *Staphylococcus aureus*, *Escherichia coli*, and *Salmonella enterica*, and therefore could also inhibit LAB, however, only in a minor way considering that LAB have the ability to synthesize a spectrum of protection compounds (polyols, peptides, and short-chain fatty acids) with a synergistic effect, counterattacking this inhibitory effect (Parmar and Rupasinghe, 2012; Zardo et al., 2020). Also, the addition of AP creates a complex profile presented by high flavor intensity, fruitiness, acidity, and the attribute of the sourdough’s typical fermentation. Synergistic effects and flavor profiles need to be future perspectives for AP in sourdough fermentation.

## Conclusion

First, AP flour had a positive effect on cell viability, with constant growth, especially in fermentations with 95% WF and 5% AP. Second, APs are a rich source of glucose and fructose, a cheap carbohydrate source for LAB and yeasts. Third, AP contains significant amounts of organic acids like oxalic acid, malic acid, and citric acid. In the context of increasing the number of by-products, it is recommended that they be used in a continuous process. Their use is imperative not to cause problems of environmental pollution. This article contributes to AP integration in sourdough fermentation and produces food enhanced with nutritious compounds, providing a better understanding of the dynamics for apple and AP to be managed and integrated into a continuous flow.

## Data Availability Statement

The original contributions presented in the study are included in the article/supplementary material, further inquiries can be directed to the corresponding author.

## Author Contributions

GM and DV developed the study concept. B-ET, GM, and FR obtained the data, conducted the analyses, and prepared the results. GM, B-ET, FR, IP, and DV contributed to the data, analyses, and interpretation. GM wrote the first draft of the manuscript with input from other authors. All authors contributed to the article and approved the submitted version.

## Conflict of Interest

The authors declare that the research was conducted in the absence of any commercial or financial relationships that could be construed as a potential conflict of interest.

## Publisher’s Note

All claims expressed in this article are solely those of the authors and do not necessarily represent those of their affiliated organizations, or those of the publisher, the editors and the reviewers. Any product that may be evaluated in this article, or claim that may be made by its manufacturer, is not guaranteed or endorsed by the publisher.
